# Predictive model for CRT risk in cancer patients with central venous access devices: a systematic review and meta-analysis

**DOI:** 10.3389/fmed.2025.1580920

**Published:** 2025-06-27

**Authors:** Wenjuan Yang, Meng Fang, Kangqin Cai, Qin Pan, Cheng Zhang, Jiquan Zhang

**Affiliations:** ^1^Department of Nephrology, Zigong First People's Hospital, Zigong, Sichuan, China; ^2^Department of Cardiovascular Medicine, Zigong First People's Hospital, Zigong, Sichuan, China; ^3^Emergency Department, Handan Central Hospital, Handan, Hebei, China; ^4^Department of Nephrology, Deyang People's Hospital, Deyang, Sichuan, China

**Keywords:** catheter-related thrombosis (CRT), central venous access devices, cancer patients, risk prediction models, meta-analysis

## Abstract

**Introduction:**

With the high incidence of central venous access device catheter-related thrombosis (CRT) in patients with cancer, its early onset, and the characteristics of clinically insignificant symptoms, risk assessment is essential for the targeted application of thromboprophylaxis. The aim of this paper was to review the risk prediction models developed for central venous access device CRT in patients with cancer and to evaluate their performance.

**Methods:**

PubMed, Embase, Web of Science, Cochrane Library, CNKI, SinoMed, Wanfang Data, and VIP databases were searched, and the search timeframes ranged from the establishment of the database to May 22, 2024. Two researchers independently performed literature screenings, data extractions, and quality assessments. The risk of bias and applicability of the included studies were assessed using the Predictive Model Risk of Bias Assessment Tool. A meta-analysis of the areas under the curve (AUC) values for model validation was performed using Stata 17.0 software.

**Results:**

Nineteen papers with 29 predictive models were included in this systematic review, reporting AUC values of 0.470–1.000. The incidence of central venous access device CRT in cancer patients ranges from 2.02 to 39.4%. The most commonly used predictors are D-dimer levels, BMI, and diabetes. All studies were judged to have a high risk of bias, mainly due to poor reporting of the areas analyzed. The combined AUC value of the six validated models was 0.81 (95% confidence interval: 0.76–0.86), indicating good model discrimination.

**Discussion:**

Most available CRT prediction models exhibited moderate-to-good predictive performance. However, all the studies were rated as having a high risk of bias according to the PROBAST scale. Future studies should adhere to methodological and reporting guidelines for large-sample, multi-center external validation of models, focusing on studies that report rigorous design and optimization or on the development of new models.

**Systematic review registration:**

PROSPERO, identifier: CRD42024516563.

## 1 Introduction

Central venous access (CVA) encompasses a range of devices, including central venous catheters (CVC), peripherally inserted central catheters (PICC), totally implantable venous access ports (TIVAP), and tunneled catheters, and play an important role in the treatment of cancer patients. CVA is widely used in systemic anticancer therapy for cancer patients, mainly in chemotherapy, blood transfusion, gastrointestinal nutrition, long-term infusion of fluids, and infusion of stimulant drugs for ambulatory patients (1). However, patients with CVA placement are at risk of developing catheter-related thrombosis (CRT), which is a common complication following the insertion of intravenous catheters (2), and is characterized by a high incidence, early onset, and insignificant clinical symptoms (3). Some studies have reported that the incidence of catheter-associated thrombosis in cancer patients is 3.6%−66% (1, 4, 5). The presence of cancer increases CRT Risk 4.1-fold (6). Catheter-associated thrombosis may lead to uncomfortable experiences such as swelling and pain in the patient's limbs (7), resulting in delayed or interrupted intravenous therapy, prolonged hospital stays, increased costs of care, unplanned extubation (8), and even pulmonary embolisms (PEs) and post-thrombotic syndrome (PTS), which may cause ongoing and progressive damage to the patient's venous function and endanger the patient's life (9, 10). Most clinical symptoms of catheter-associated thrombosis are not apparent. As a result, methods to prevent these events have gained significant attention from oncologists, encouraging specialists to prioritize this issue.

Central venous CRT in patients with cancer often results from a combination of multiple risk factors, and the clinical knowledge of CRT prevention is limited to experience and imaging. Although prophylactic anticoagulation reduces the incidence of CRT in cancer patients, it increases the risk of bleeding (11, 12). Most current international guidelines do not recommend routine anticoagulation therapy alone to prevent the need for CRT (13, 14). Therefore, it is important to assess the risk of catheter-associated thrombosis according to the conditions of cancer patients to help clinical staff identify the high-risk group for central venous CRT in cancer patients at an early and precise stage and to identify the risk factors for central venous catheter-associated thrombosis to predict the occurrence of CRT (15), which is conducive to the timely implementation of targeted prevention and treatment in the clinic to reduce the incidence of CRT. Risk prediction models use quantitative research methods to predict morbidity risk more accurately and present findings using more intuitive data (16). Predictive models can help healthcare professionals identify the risk of cancer patients developing central venous CRT at early stages and improve early warning awareness.

Several prediction models have been developed for CRT risk in patients with cancer; however, there are differences in the quality of the studies and large gaps in predictive performance. To date, no studies have summarized the quality and applicability of these published models, nor have they analyzed and compared their predictive performance. Therefore, by systematically evaluating the risk of bias and applicability of CRT risk prediction models for cancer patients, the present study aimed to help select the best prediction model for clinical practice and provide a valuable reference for constructing high-performance risk prediction models for future studies.

## 2 Methods

This study followed the general principles recommended in the Preferred Reporting Items for Systematic Reviews and Meta-Analyses (PRISMA) statement for systematic reviews and meta-analyses (17). The study protocol was registered with PROSPERO (registration number: CRD42024516563).

### 2.1 Review questions

In this systematic review, we followed the modified PICOTS system to formulate the following review question (18): Which models are available at this stage for predicting the risk of CRT in patients with cancer? What is the validity and utility of these models? The PICOTS statement is as follows.

(1) P (Population): The population of interest comprises cancer patients who aged above 18 years old.
(2) I (Index model): All available central venous CRT risk prediction models.
(3) C (Comparator model): not applicable.
(4) O (Outcome): The outcome was defined as the occurrence of CRT. All diagnostic criteria adopted by the studies were accepted.
(5) T (Timing): Predicted application time after central venous catheterization in patients with cancer.
(6) S (Setting): Risk prediction model applied to cancer patients with CVA devices.

### 2.2 Eligibility criteria

Inclusion Criteria: (1) Population: cancer patients aged ≥18 years who underwent central venous catheterization; (2) Type of study: cohort, case-control, cross-sectional; and (3) Study content: a study on the construction of a prediction model for the risk of catheter-associated thrombosis due to the placement of indwelling CRT in cancer patients and a description of the process of constructing the prediction model. The exclusion criteria were as follows: (1) studies without predictive models; (2) articles in languages other than Chinese or English; (3) informally published literature, such as conference abstracts and dissertations; (4) models constructed on the basis of systematic evaluation/meta-analysis; (5) models with < 2 predictors, Single-predictor models may lack robustness and clinical utility; (6) studies that tested risk assessment scales; and (7) authors contacted by email but failed to retrieve the full text.

### 2.3 Search strategy

Considering the large population size and the universality of the languages, we conducted a comprehensive search of both Chinese and English databases. English databases, including PubMed, Embase, Web of Science, Cochrane Library, Chinese databases, China Knowledge (CNKI), China Biomedical Literature Service System (SinoMed), Wanfang Database, and Wipro Chinese Science and Technology Journal Database (VIP), were searched for studies on CRT in cancer patients. For the risk prediction model, the search strategy was conducted using a combination of subject terms and free words, including Medical Subject Headings (MeSH) terms and keywords related to cancer, central venous catheter, thrombosis, catheter-associated thrombosis, risk prediction model, and risk factors. Moreover, the search was conducted mainly by computer, then supplemented by manual search and further traced by searching the references of the selected papers. The search timeframe was from the establishment of the database to May 22, 2024. The detailed search strategies are provided in the Supplementary material.

### 2.4 Study selection and data extraction

Two researchers independently screened the results of the literature search according to the inclusion and exclusion criteria. If there was a difference of opinion between the two and no consensus could be reached after discussion, a third party opinion was sought and agreed upon. As for the literature screening method, after removing duplicates using the EndNote software, duplicate titles were manually removed, titles and abstracts were read for initial screening, and after excluding obviously irrelevant literature, the full text was further read for rescreening to determine the final included literature. After determining the included literature, we developed a standardized form for use in systematic reviews of prediction modeling studies based on the Critical Appraisal and Data Extraction for Systematic Reviews of Prediction Modeling Studies (CHARMS) (19). The extracted data included (1) the basic characteristics and predictive results of the included literature, such as basic information of authors, year of publication, country of publication, study design, study population, method of catheter placement, data sources, and predictive results; and (2) the construction of a predictive model of CRT risk in cancer patients, such as the number of candidate variables, continuous variable processing methods, predictor screening methods, model building methods, model validation methods, model performance, model calibration methods, predictors, and model presentation method.

### 2.5 Study quality assessment

Two researchers independently used the Risk of Bias and Applicability Assessment Tool for Predictive Modeling Studies (PROBAST) (20) to assess the risk of bias and applicability of the models included in the literature. For the risk of bias assessment, PROBAST classified the potential bias involved in predictive modeling studies into 4 domains—namely, study population, predictors, outcomes, and analyses, which contained 2, 3, 6, and 9 questions, respectively, for a total of 20 questions. Evaluators made judgments based on the literature for each question, with responses to each question including “yes,” “probably yes,” “no,” “probably no,” or “no information.” The risk of bias for the prediction model as a whole and for each domain was categorized as low, high, or unclear. The bias risk was considered “low risk” when all questions in the area were answered “yes” or “probably yes.” The risk was “high” when one question was answered “no” or “probably no.” When an issue is deemed to have “no information” and all other issues are “low risk,” the area is classified as “unclear.” For the suitability assessment, the evaluation of the suitability of the prediction model contained three domains: study object, predictor, and outcome. The judgment process was similar to the risk of bias. The overall applicability of the forecasting model was rated as “low,” “high,” and “unclear.” Only when all areas were “low risk” was the whole judged to be “low risk.” If one or more areas were judged to be “high risk,” the whole was classified as “high risk.” If an area was judged to be “unclear” and all other areas were “low risk,” the overall classification was “unclear.”

### 2.6 Statistical analysis

The features of the included CRT risk-prediction models were collated and synthesized to compare model discrimination and calibration. Meta-analysis of the area under the curve (AUC) values of the validated model was performed using Stata 17 software. The *I*^2^ statistic was used to assess the degree of heterogeneity. The *I*^2^ index provided a measure of heterogeneity, with values of 25 percent, 50 percent, and 75 percent indicating low, medium, and high heterogeneity, respectively (21). The choice to use a fixed- or random-effects model was based on the heterogeneity of the analyzed results.

## 3 Results

### 3.1 Study flow

**Figure 1:**
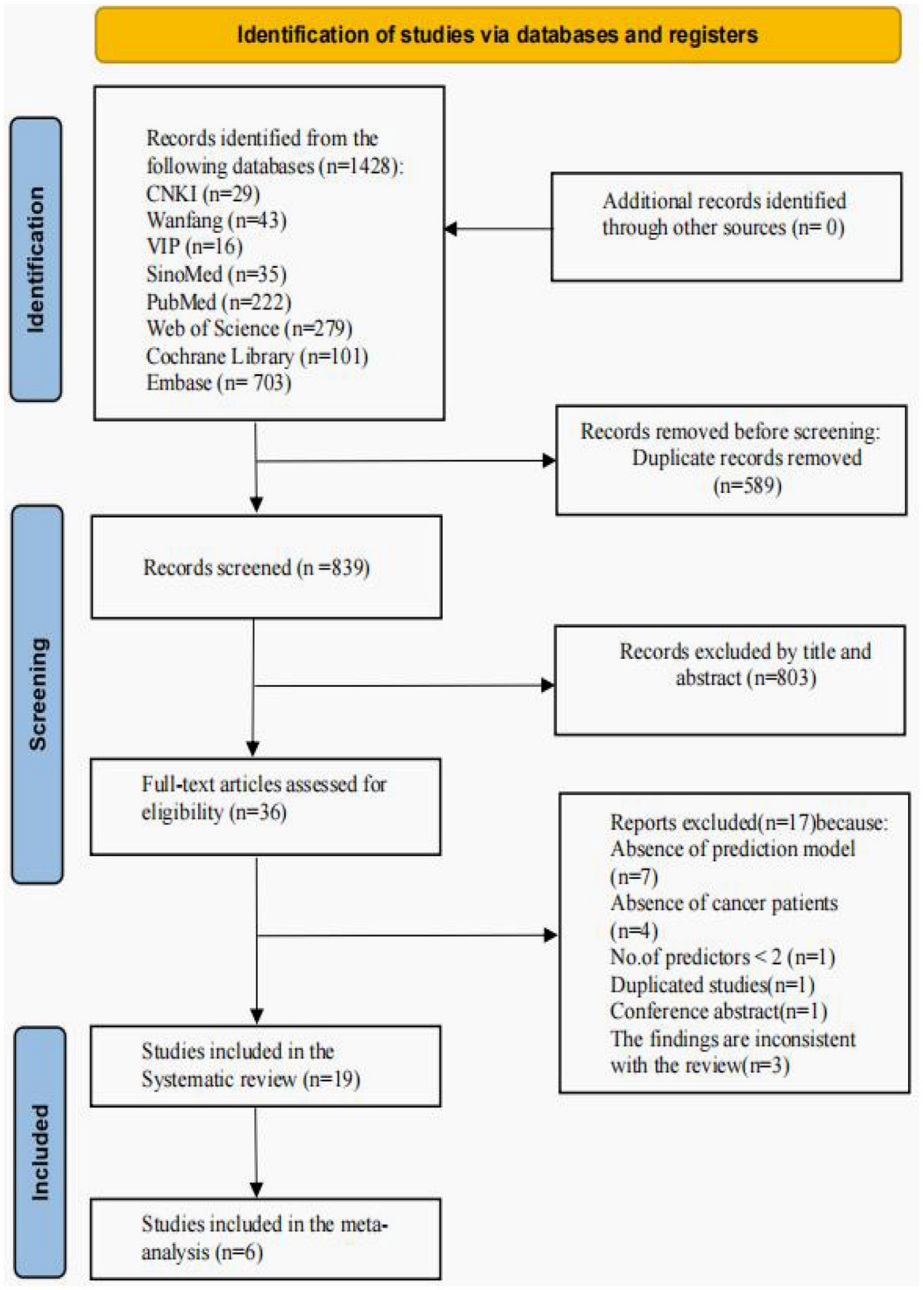
PRISMA flowchart of literature search and selection.

The initial search yielded 1,428 records, of which 589 duplicate records were removed from all databases. A total of 803 documents were screened by reading their titles and abstracts, leaving 36 documents for further screening. Of these 36 papers, 7 studies were excluded because they did not develop predictive models or only performed risk factor analyses, 4 included studies that included non-cancer patients, 1 had < 2 predictors, 3 had outcomes that were inconsistent with the content of the review, 1 was a duplication of a study, and 1 was a conference abstract. Finally, 19 studies with 29 models were included (Figure 1).

### 3.2 Study characteristics

Nineteen studies were included in this review; 18 studies (22–39) were conducted in China, and 1 study (40) was conducted in Israel, published from to 2018–2024. A total of 20,691 patients were included in the study, and the total sample size of all studies ranged from 286 to 5091 patients. Two studies (22, 38) were case-control studies; four studies (23, 25, 37, 39) were cross-sectional studies; five studies (24, 26, 30, 32, 36) were prospective cohort studies; seven studies (27–29, 31, 33, 40) were retrospective studies, and one study (34) used retrospective cohort studies to collect patient-related clinical data when constructing the model. This validated the constructed model on two occasions, respectively, and the validation set data were collected using prospective cohort studies and retrospective cohort studies for data collection, respectively. Three studies (23–25) were conducted on patients with lung cancer; one (28) on patients with hematological malignancies; one (29) on patients treated with chemotherapy for tumors; one (40) on patients with newly diagnosed acute myeloid leukemia; two (32, 33) on patients with breast cancer; one (39) on patients with lymphoma; and ten studies (22, 26, 27, 30, 31, 34–37, 39) on patients with various types of cancer. One study (29) used an implantable venous access port; 4 studies (22, 31, 34, 40) used CVC; and 14 studies (23–28, 30, 32, 33, 35–39) used peripherally placed central venous catheter placement. Four studies (22, 31, 34, 40) predicted the outcome of CVC-associated thrombosis; eight studies (24, 25, 27, 28, 32, 33, 35, 36) predicted PICC catheter-related thrombosis; four studies (23, 26, 30, 37) predicted PICC-related upper extremity deep vein thrombosis; and two studies (38, 39) predicted PICC-related venous thrombosis. One study reported an infusion port-associated thrombosis (29). All participants were cancer patients recruited from 2006 to 2023, of whom 1,928 developed central venous CRT, with an overall incidence of 2.02%−39.4% (Table 1).

**Table 1:** Characteristics of included studies.

**Author, year**	**Country**	**Period**	**Study design**	**Participants**	**Age, years**	**Method of catheter placement**	**Results of prediction**	**Total participants (*N*)/Events (*N*)**	**Incidence (%)**	**Duration of follow up**
Wang, He, Chu, Chen, and Wang (22)	China	January 2019 to June 2022	Case-control study	Malignant tumor	7–86 (54.00 ± 11.83)	CVC	CRT	2,096/178	8.5%	—
Dong, Zhang, Zhu, Sun, Xi, Li, and Gu (23)	China	September 2019 to October 2021	Cross-sectional study	Lung cancer	UEDVT group: 58.2 ± 9.4 non-UEDVT group: 56.7 ± 7.6	PICC	UEDVT	296/51	17.2%	—
Gao, Zhao, Yang, Sun, Hua, Wu, Wang, Gu, Zhou, and Bi (24)	China	January 2016 to December 2019	Prospective study	Lung cancer	CRT group: 41–80 (61.64 ± 8.35) Non-CRT group: 23–85 (61.11 ± 8.81)	PICC	CRT	5,091/103	2.02%	—
Huang, Chen, Deng, Shi, and Shang (25)	China	May 2019 to May 2022	Cross-sectional study	Lung cancer	43–84 (62.13 ± 9.73)	PICC	CRT	453/48	10.59%	—
Sun, Song, Zhang, and Song (26)	China	April 2016 to December 2019	Prospective cohort study	Malignant tumor	≥18	PICC	UEDVT	357/38	10.6%	—
Zhang, Xie, Zhou, and Hao (27)	China	December 2014 to December 2015	Retrospective study	Malignant tumor	CRT group: (53.26 ± 13.88) Non-CRT group: (55.40 ± 12.66)	PICC	CRT	286/72	25.17%	—
Zhou, Wang, Lu, Zhou, Liu, Dong, and Li (28)	China	January 2019 to December 2020	Retrospective study	Hematological malignant tumor	CRT group: (51.77 ± 14.26) Non-CRT group: (53.88 ± 15.06)	PICC	CRT	980/53	5.41%	—
Chen, Zhang, Li, Shi, and Gon (29)	China	January 2015 to December 2018	Retrospective study	Tumor chemotherapy	19–82 (56.44 ± 10.83)	IVAP	CRT	Derivation cohort: 372/25 Validation cohort: 2,48/15	Derivation cohort:6.72% Validation cohort: 6.05%	—
Yang, Hua, Wu, Bi, Wu, Wang, Gao, Liang, and Wu (30)	China	January 2016 to March 2017	Prospective cohort study	Malignant tumor	(56 ± 11)	PICC	UEDVT	1,032/26	2.52%	Follow-up at the end of tube insertion, 1 week, 2 weeks, 1 month, 3 months, 6 months, 9 months, and at the time of extubation
Lin, Zhu, YihanZhang, Du, and Zhang (31)	China	January 2019 to December 2020	Retrospective study	Cancer	Derivation cohort:(60.50 ± 8.34) Validation cohort:(61.12 ± 9.07)	CVC	CRT	Derivation cohort: 431/166 Validation cohort: 216/85	Derivation cohort:38.5% Validation cohort: 39.4%	—
Fu, Cai, Zeng, He, Bao, Lin, Lin, Hu, Lin, Huang, Zheng, Chen, Zhou, Lin, and Fu (32)	China	January 2018 to June 2021	Prospective cohort study	Breast cancer	22–77 Derivation cohort:(47.8 ± 9.2) Validation cohort:(48.7 ± 9.0)	PICC	CRT	1,844/256	13.9%	—
Peng, Wei, Li, Yuan, and Lin (33)	China	January 1, 2015, to August 31, 2019	Retrospective study	Breast cancer	Median 47 (IQR 42, 53)	PICC	CRT	Derivation cohort: 978/40 Validation cohort: 284/10	Derivation cohort: 4.09% Validation cohort: 3.52%	—
Liu, Xie, Sun, Wang, Yuan, Liu, Huang, Wang, Mo, Yi, Guan, Li, Wang, Li, Ma, and Zeng (34)	China	January 1, 2015 to December 31, 2018	Derivation cohort: Retrospective cohort study Validation cohort 1: Prospective cohort study Validation cohort 2: Retrospective cohort study	Malignant tumor	Derivation cohort:(53.7 ± 11.1) Validation cohort 1:(54.2 ± 11.7) Validation cohort 2:(59.2 ± 11.0)	CVC	CRT	3,131/397	12.7%	CVC followed up to 3 months PICC follow-up to 12 months
Song, Lu, Chen, Bao, Li, Li, Peng, Liu, Chen, Li, and Zhang (35)	China	Since January 2018, in 10 months	Retrospective study	Cancer	CRT group: (56.76 ± 13.21) Non-CRT group: (52.95 ± 14.57)	PICC	CRT	339/59	17.4%	—
Liu, Zhang, Xie, Wang, Xiang, Yue, Feng, Yang, Li, Luo, and Yu (36)	China	February 1, 2016, to February 31, 2017	Prospective cohort study	Cancer	NR	PICC	CRT	348/57	16.38%	Follow-up 30 days after catheter insertion
Hu, Wu, and Zhao (37)	China	April 2021 to December 2021	Cross-sectional study	Malignant tumor	≥18	PICC	UEDVT	452/76	16.8%	Routine ultrasound examinations were performed weekly for 4 weeks starting on the day of tube insertion
Wang, He, Chu, and Xu (38)	China	Derivation cohort: January 2017 to December 2020 Validation cohort: January 2021 to September 2023	Retrospective case-control study	Patients with lymphoma	Derivation cohort:(52.43 ± 14.94) Validation cohort:(51.43 ± 13.89)	PICC	RVTE	305/35	11.48%	—
Du, and Din (39)	China	Derivation cohort: March 2017 to May 2021 Validation cohort: June 2021 to February 2023	Cross-sectional study	Elderly patients with cancer	Derivation cohort:(71.12 ± 4.20) Validation cohort:(71.23 ± 3.42)	PICC	RVTE	Derivation cohort: 400/74 Validation cohort:120/—	Derivation cohort: 18.5% Validation cohort:—	—
Perek, Khatib, Izhaki, Khalaila, Brenner, and Horowitz (40)	Israel	Between 2006 and 2019	Retrospective study	Patients with newly-diagnosed acute myeloid leukemia	CRT group: (57.4 ± 14.0) Non-CRT group: (54.6 ± 15.7)	CVC	CRT	632/64	10.1%	From tube insertion to the occurrence of CRT, death, or final follow-up. The date of final follow-up is 1 December 2019

CVC, Central venous catheter; PICC, Peripherally inserted central venous catheter; IVAP, Implantable venous access port; CRT, catheter-related thrombosis; UEDVT, Upper extremity deep vein thrombosis; RVTE, Related Venous Thrombosis.

### 3.3 Model development and performance

A total of 19 studies were included in this review, reporting 29 predictive models, of which 4 studies (26, 37, 39, 40) reported 2 models and 2 studies (32, 36) reported 4 models. Thirteen studies (23–25, 27–34, 36, 40) reported the number of candidate factors included, with quantities ranging from 19 to 39, whereas the remaining studies did not. Seventeen studies (22–25, 27–35, 37–40) reported the treatment of continuous variables, and seven studies (24, 27, 29, 30, 33, 34, 39) treated continuous variable data as categorical variables. Only two studies (31, 34) reported the direct deletion of missing data. Twelve studies (22, 24–31, 33, 36, 37) (63.2%) reported model development and internal validation; two studies (32, 39) (10.5%) reported model development and temporal validation; one study (38) (5.3%) reported model development and geographic validation; and one study (34) (5.3%) conducted both temporal and geographical validations. However, there are still three studies (23, 35, 40) (15.8%) that developed models without validation. In terms of variable screening, four studies (26, 27, 36, 37) used the least absolute shrinkage and selection operator (LASSO) regression analysis, and six studies (24, 32–35, 40) used stepwise, forward stepwise, backward stepwise, forward, and backward stepwise analyses for variable screening. Twelve studies (22–25, 28, 29, 31, 33–35, 38, 40) (68.4%) used logistic regression analysis to build the model; one study (30) used Cox regression analysis; five studies (26, 32, 36, 37, 39) applied machine learning (ML) to build the predictive model; and one study (27) did not report model-building methods. In terms of model validation, six studies (22, 24, 25, 27, 30, 31) used bootstrapping for internal validation, two studies (36, 37) used cross-validation, and four studies (26, 28, 29, 33) were randomly assigned according to proportions. Four studies (23, 35, 38, 39) used external validation [2 (32, 39) used temporal validation, one (34) performed temporal and geographic validation, and one (38) used geographic validation]. The AUC was the most commonly used method for evaluating discrimination, with reported AUC values ranging from 0.470 to 1.000. Three studies reported C-indices ranging from 0.688 to 0.824. In terms of model calibration, eight studies (22, 23, 25, 29, 33, 34, 38, 40) used the Hosmer-Lemeshow test, four studies (22, 23, 25, 29) reported Hosmer-Lemeshow test *P*-values, and nine studies (22–25, 27, 30, 31, 34, 38) provided calibration plots. However, seven studies (26, 28, 32, 35–37, 39) did not report calibration performance. Thirteen studies reported categorical indicators, such as sensitivity and specificity, with reported sensitivities ranging from 50.0 to 100% and specificities ranging from 54.8 to 100%. In terms of model presentation, 10 studies (22–25, 27, 30, 31, 35, 38) provided the final nomogram model, 2 studies (28, 33) derived risk score formulas based on partial regression coefficients for each factor for presentation, 1 study (29) provided a demonstration of the model as a scoring system, and the remaining 6 studies (26, 32, 36, 37, 39, 40) did not report model presentation (Table 2).

**Table 2:** Overview of the information of the included prediction models.

**Author, year**	**Number of candidate variables**	**Continuous variable processing method**	**Missing data handling**	**Variable selection**	**Modeling method**	**Calibration method**	**Validation method**	**Model performance (95%CI if reported)**	**Risk factors included in models (*n*)**	**Model presentation**
Wang, He, Chu, Chen, and Wang (22)	—	Continuous variable	—	—	LR	H-L *P* = 0.773 Calibration plot	Internal validation Bootstrap	Derivation cohort: AUC = 0.856 (0.824–0.889) Sensitivity:79.00% Specificity:74.00% C-index: 0. 824 Validation cohort: AUC— Sensitivity:—Specificity:—	(6) TNM stage Co-infection Using hormones History of thrombosis/ hypercoagulability HPD-dimer	Nomogram
Dong, Zhang, Zhu, Sun, Xi, Li, and Gu (23)	21	Continuous variable	—	—	LR	H-L *P* = 0.565 Calibration plot	—	Derivation cohort: AUC = 0.787 (0.718–0.856) Sensitivity:86.3% Specificity:89.4% Validation cohort: AUC— Sensitivity:—Specificity:—	(5) Diabetes TNM stage > II Catheter end position in the upper 2/3 of the superior vena cava Catheter retention time Plasma D-dimer	Nomogram
Gao, Zhao, Yang, Sun, Hua, Wu, Wang, Gu, Zhou, and Bi (24)	37	Categorical variables	—	Forward stepwise analysis	LR	Calibration plot	Internal validation Bootstrap	Derivation cohort: AUC = 0.794 (0.744–0.845) Sensitivity:64.3% Specificity:85.2% Accuracy:80.8% Validation cohort: AUC— Sensitivity:—Specificity:—	(10) History of PICC catheter Tube feeding times ≥ 2 With other catheter-related complications High LDL cholesterol Secondary catheter ectasia TNM Stage IV Venous compression Smoking history D-dimer Barthel score < 80	Nomogram
Huang, Chen, Deng, Shi, and Shang (25)	19	Continuous variable	—	—	LR	H-L *P* = 0.437 Calibration plot	Internal validation Bootstrap	Derivation cohort: AUC = 0.823 (0.767–0.879) Sensitivity:79.2% Specificity:69.9% Validation cohort: AUC— Sensitivity:—Specificity:—	(7) Staging of lung cancer before tube placement Sex Pre-intubation VTE score Pre-intubation mobility score History of cerebral infarction History of cancer metastasis Pre-intubation D-dimer	Nomogram
Sun, Song, Zhang, and Song (26)	—	—	—	LASSO regression method	ML methods	—	Internal validation split sample 5:5	ML-LASSO Derivation cohort: AUC— Sensitivity:96.6% Specificity:87.4% Testing cohort: AUC = 0.856 (0.782–0.931) Sensitivity:76.2% Specificity:72.1% ML-Seeley-LASSO Derivation cohort:— Sensitivity:100% Specificity:100% Testing cohort: AUC = 0.799 (0.711–0.887) Sensitivity:54.3%Specificity:54.9%	ML-LASSO (4) BMI (10.32 points) Smoking (7.51 points) Family history of DVT (6.30 points) NR2002 Score (4.11 points) ML-Seeley-LASSO (4) Diabetes (9.08 points) BMI(8.2 points) Catheter location (5.27 points) D-dimer (≥0.5 mg/L; 3.09 points)	—
Zhang, Xie, Zhou, and Hao (27)	27	Categorical variables	—	LASSO regression method	—	Calibration plot	Internal validation Bootstrap	C-index:0.688	(4) D-dimer Peripheral vascular puncture without ultrasound guidance Chemotherapy history Presence of other comorbidities during intubation	Nomogram
Zhou, Wang, Lu, Zhou, Liu, Dong, and Li (28)	39	Continuous variable	—	—	LR	—	Internal validation split sample 7:3	Derivation cohort: AUC = 0.786 (0.719–0.852) Sensitivity:82.9% Specificity:64.3% Validation cohort: AUC = 0.856 (0.735–0.978) Sensitivity:80.0%Specificity:90.0%	(6) Sex Recombinant Human Granulocyte Colony-stimulating Factor APTT D-dimer TAGHB	Formula of risk score obtained by partial regression coefficient of each factor
Chen, Zhang, Li, Shi, and Gon(29)	30	All variables except the number of chemotherapy sessions and indwelling time were treated as dichotomous variables	—	—	LR	H-L *P* = 0.347	Internal validation split sample 6:4	Derivation cohort: AUC = 0.748 (0.707–0.873) Sensitivity:— Specificity:— Validation cohort: AUC = 0.837 (0.749–0.925) Sensitivity:—Specificity:—	(5) Number of chemotherapy sessions TNM stage Thrombotic history D-dimer History of ipsilateral CVC	Scoring system
Yang, Hua, Wu, Bi, Wu, Wang, Gao, Liang, and Wu (30)	32	Categorical variables	—	—	COX regression	Calibration plot	Internal validation Bootstrap	C-index:0.71 (0.630–0.800)	(2) Thrombotic historyHP	Nomogram
Lin, Zhu, Yihan Zhan, Du, and Zhang (31)	36	Continuous variable	Deletion	—	LR	Calibration plot	Internal validation Bootstrap	Derivation cohort: AUC = 0.757 (0.717–0.809) Sensitivity:— Specificity:— Validation cohort: AUC = 0.761 (0.701–0.821) Sensitivity:—Specificity:—	(4) BMI Cancer types D-dimerBlood_flow_velocity	Nomogram
Fu, Cai, Zeng, He, Bao, Lin, Lin, Hu, Lin, Huang, Zheng, Chen, Zhou, Lin, and Fu (32)	19	Continuous variable	—	Stepwise Regression Analysis (Logistic regression method)	ML methods ANNLR	—	Temporal validation	Using SMOTE Derivation cohort (ANN): AUC = 0.742 Sensitivity:72.7% Specificity:71.2% Accuracy:71.5% Derivation cohort (LR): AUC = 0.675 Sensitivity:76.4% Specificity:58.9% Accuracy:61.7% No SMOTE Derivation cohort (ANN): AUC = 0.725 Sensitivity:80.0% Specificity:61.6% Accuracy:64.6% Derivation cohort (LR): AUC = 0.670 Sensitivity:76.4% Specificity:59.3%Accuracy:61.7%	(8) Age Comorbidities Upper extremity activity CVC history Education status Chemotherapy status Insertion attemptsLaterality	—
Peng, Wei, Li, Yuan, and Lin (33)	29	Categorical variables	—	Stepwiseselection	LR	H-L	Internal validation split sample 3:1	Derivation cohort: AUC = 0.850 (0.776–0.924) Sensitivity:75.0% Specificity:83.2% Validation cohort: AUC = 0.882 (0.781–0.984) Sensitivity:70.0%Specificity:84.7%	(9) CVC History COPD PLT D-dimer APTT Menopause Breast surgery Upper extremity lymphedema Endocrine therapy	Formula of risk score obtained by partial regression coefficient of each factor
Liu, Xie, Sun, Wang, Yuan, Liu, Huang, Wang, Mo, Yi, Guan, Li, Wang, Li, Ma, and Zeng (34)	30	Categorical variables	Deletion	Stepwise forward and backward selection	LR	H-L Calibration plot	Temporal validation Geographical validation	Derivation cohort: AUC = 0.741 (0.715–0.766) Sensitivity:67.3% Specificity:69.8% Validation cohort 1:AUC = 0.754 (0.704–0.803) Sensitivity:— Specificity:— Validation cohort 2: AUC = 0.658 (0.470–0.845) Sensitivity:—Specificity—	(6) Sex Cancer types Catheter type Position of the catheter tip Chemotherapy status Antiplatelet/ Anticoagulation status	Nomogram
Song, Lu, Chen, Bao, Li, Li, Peng, Liu, Chen, Li, and Zhang (35)	—	Continuous variable	—	Stepwise regression analysis	LR	—	—	Derivation cohort: AUC = 0.812 (0.749–0.875) Sensitivity:74.9%Specificity:64.4%	(4) Height D-dimer Puncture times Patient's performance status	Nomogram
Liu, Zhang, Xie, Wang, Xiang, Yue, Feng, Yang, Li, Luo, and Yu (36)	38	—	—	LASSO regression method	ML methods	—	Internal validation Monte Carlo cross-validation	Seeley-LASSO-RF Derivation cohort: AUC = 0.876 Sensitivity:82.8% Specificity:88.4% Testing cohort:AUC = 0.798 Sensitivity:75.0% Specificity:83.5% Seeley-RF Derivation cohort: AUC = 1.00 Sensitivity:100% Specificity:100% Testing cohort:AUC = 0.729 Sensitivity:46.4% Specificity:93.8% RF Derivation cohort: AUC = 1.00 Sensitivity:100% Specificity:100% Testing cohort: AUC = 0.775 Sensitivity:50.0% Specificity:88.3% LASSO-RF Derivation cohort: AUC = 0.936 Sensitivity:82.8% Specificity:97.3% Testing cohort: AUC = 0.809 Sensitivity:90.3%Specificity:71.4%	Seeley-LASSO-RF (8) Seeley (15.79 points) Drinking (13.96 points) NRS 2002 Score (8.45 points) Family History of DVT (6.36 points) Diabetes (4.91 points) Malposition (3.35 points) Tip Location (3.3 points) Chemotherapy (2.06 points) Seeley-RF (8) Genotype (10.61 points) Sex (10.06 points) Age (6.92 points) BMI (6.73 points) Smoking (5.12 points) Drinking (5.07 points) NRS 2002 Score (4.91 points) Self-care Ability Score (4.1 points) RF (8) Genotype (14.08 points) Sex (7.13 points) Age (7.07 points) BMI (5.75 points) Smoking (5.7 points) Drinking (5.58 points) NRS 2002 Score (3.99 points) Self-care Ability Score (3.87 points) LASSO-RF (8) Drinking (24.6 points) NRS2002 Score (10.87 points) Family history of DVT (7.22 points) Diabetes (5.83 points) Tip Location (4.14 points) Chemotherapy Cycle (2.93 points) Radiotherapy (2.49 points) Anticoagulant therapy (2.25 points)	—
Hu, Wu, and Zhao (37)	—	Continuous variable	—	LASSO regression method	ML methods	—	Internal validation Cross-validation	ML-LASSO Derivation cohort: AUC = 0.855 (0.781–0.932) Sensitivity:96.5% Specificity:87.4% Testing cohort:AUC = 0.856 Sensitivity:76.2% Specificity:72.1% ML-Seeley-LASSO Derivation cohort: AUC = 0.798 (0.712–0.886) Sensitivity:100% Specificity:100% Testing cohort: AUC = 0.799 Sensitivity:54.2%Specificity:54.8%	ML-LASSO (4) BMI (10.31 points) Smoking (7.52 points) Family history of DVT (6.32 points) NRS2002 Score (4.13 points) ML-Seeley-LASSO (4) Diabetes (9.09 points) BMI (8.1 points) Catheter displacement (5.23 points) D-dimer (≥0.5 mg/L; 3.06 points)	—
Wang, He, Chu, and Xu (38)	—	Continuous variable	—	—	LR	H-L Calibration plot	Geographical validation	Derivation cohort: AUC = 0.907 (0.850–0.964) Sensitivity:— Specificity:— Validation cohort: AUC= 0 .896 (0.782–1.000) Sensitivity:—Specificity:—	(6) Activity amount Thrombosis history (within the last 12 months) Antithrombin III Position of catheter tip Total cholesterol D-dimer levels	Nomogram
Du, and Din (39)	—	All variables except age were treated as categorical variables	—	—	ML methods: LRDT	—	Temporal validation	Derivation cohort:(LR):AUC = 0.701 (0.633–0.770) Sensitivity:— Specificity:— Derivation cohort:(DT):AUC = 0.749 (0.688–0.811) Sensitivity:— Specificity:— Validation cohort(DT):AUC = 0.812 (0.783–0.841) Sensitivity:—Specificity:—	(5) BMI Puncture times Catheter retention time Diabetes Chronic renal insufficiency	—
Perek, Khatib, Izhaki, Khalaila, Brenner, and Horowitz (40)	31	Continuous variable	—	Backward stepwiseselection	LR	H-L	—	Model 1: Derivation cohort: AUC = 0.698 (0.626–0.771) Sensitivity:76.0% Specificity:56.0% Model 2: Derivation cohort: AUC = 0.711 (0.635–0.789) Sensitivity:61.0%Specificity:72.0%	Model 1: (4) Prior history of venous thromboembolism Acute promyelocytic leukemia BMI Initial platelet counts Model 2: (4) BMI Prior history of venous thromboembolism COPD Initial platelet count	—

“—,” Not reported; LASSO, The least absolute shrinkage and selection operator; LR, Logistic regression; R, The R Programming Language; ML, Machine learning; DT, Decision tree; H-L, Hosmer-Lemeshow; AUC, Area under curve; SMOTE, Synthetic minority oversampling technique; ANN, Artificial Neural Network; TNM, Tumor Node Metastasis; PICC, Peripherally Inserted Central Catheter; LDL, Low-density lipoprotein; VTE, Venous thromboembolism; BMI, Body mass index; NR2002, Nutrition Risk Screening 2002; APTT, Activated partial thromboplastin time; TAG, Triacylglycerol; HB, Hemoglobin; HP, Hypertriglyceridemia; CVC, Central venous catheter; COPD, Chronic obstructive pulmonary disease; PLT, Platelet Levels; DVT, Deep venous thrombosis; RF, Random forests.

### 3.4 Predictors included in the models

**Figure 2:**
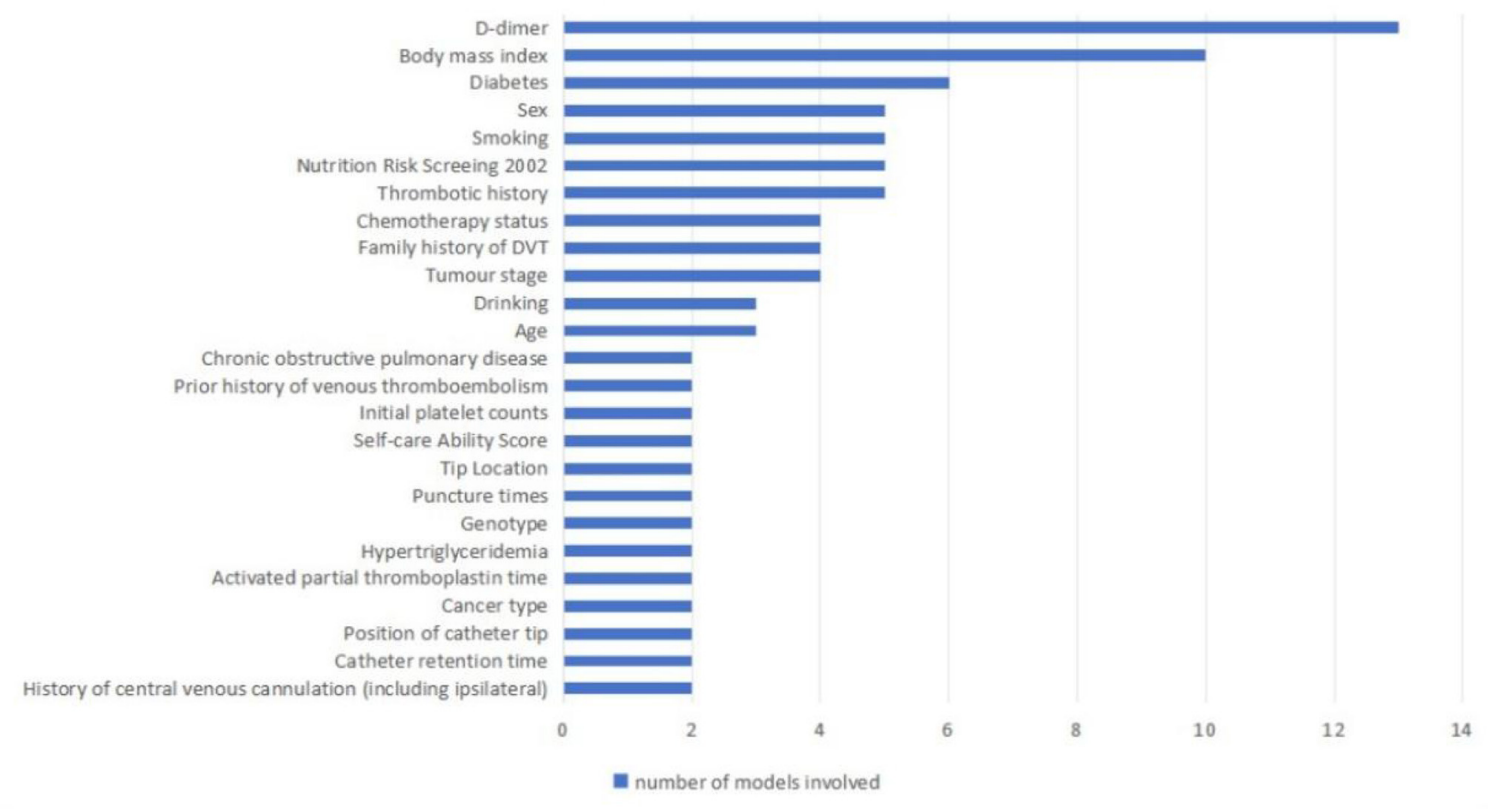
Risk factors included in the CRT prediction model.

Candidate factors considered in developing predictive models for CRT in patients with cancer include individual patient-related factors, the disease itself, disease treatment factors, catheter placement factors, and laboratory index-related factors. The final number of predictors retained in the model ranged from 2 to 10. The predictors, in order of most common to least common, were D-dimer (*n* = 13), Body mass index (*n* = 10), Diabetes (*n* = 6), Sex (*n* = 5), Smoking (*n* = 5), Nutrition Risk Screening 2002 (*n* = 5), Thrombotic history (*n* = 4), Chemotherapy status (*n* = 4), Family history of DVT (*n* = 4), Tumor stage (*n* = 4), Drinking (*n* = 3), Age (*n* = 3), Chronic obstructive pulmonary disease (*n* = 2), Prior history of venous thromboembolism (*n* = 2), Initial platelet counts (*n* = 2), Self-care Ability Score (*n* = 2), Tip Location (*n* = 2), Puncture times (*n* = 2), Genotype (*n* = 2), Hypertriglyceridemia (*n* = 2), Activated partial thromboplastin time (*n* = 2), Cancer type (*n* = 2), Position of catheter tip (*n* = 2), Catheter retention time (*n* = 2), and History of central venous cannulation (including ipsilateral; *n* = 2; Table 2, Figure 2).

### 3.5 Results of quality assessment

The risk of bias was evaluated for the 19 included studies based on the PROBAST evaluation criteria, and it was found that all included studies were at high risk of bias. Regarding the study population, after evaluation, 12 studies (22, 23, 25, 27, 28, 31, 33, 38–40) were rated as high risk, 5 studies (24, 26, 30, 32, 34) as low risk, 2 studies (35, 36) were rated as unclear because information related to the inclusion and exclusion criteria was not reported, and 8 studies (23, 25, 27, 29, 30, 35, 37, 39) could not be discerned from the original text as to whether patients already suffering from CRT were included. The inclusion of patients who already had a confirmed CRT diagnosis may have led to a higher rate of false positives in the prediction model. In terms of predictors, six studies (22, 26, 29, 30, 34, 35) had a high risk of bias, nine studies (24, 27, 28, 31, 32, 36, 38, 40) had a low risk of bias, and the risk of bias for four studies (23, 25, 37, 39) was rated as unclear. When assessing predictors, measurements may be assessed by different healthcare professionals in the clinic, which may result in an increased risk of predictor domains. One study (34) collected data in three healthcare organizations, but differences in the methods of assessing predictors and the assessors in different healthcare organizations may result in biased results in the assessment of predictors (41). Therefore, for the question “Are the definition and assessment of the predictors the same for all subjects,” studies indicating that they were assessed by the researcher himself or by uniformly trained medical and subject personnel were rated “Yes.” Included studies that did not state the qualifications of the person assessing the predictor were rated as “probably no.” The outcome indicator “CRT” requires vascular color Doppler ultrasound, angiography, or other ancillary tests for diagnosis and does not affect the assessment of predictors. Therefore, the question “whether the predictors were assessed without knowing the outcome data” was rated as “yes” for prospective studies and “probably yes” for retrospective studies. In terms of outcome, the risk of bias was unclear in 2 studies (30, 33), high in 6 studies (22, 27, 29, 35–37), and low in 11 studies (23–26, 28, 31, 32, 34, 38–40). Three studies (22, 33, 40) used machine learning methods to construct CRT risk prediction models, some of which included the Seeley scale (Seeley et al. developed a predictive tool for predicting upper extremity venous thrombosis) (42), which may have led to an overestimation of the association between the predictors and outcomes of the model. Three studies (27, 29, 30) did not provide details on the method of determining outcomes and lacked information on the appropriate determination of clinical outcomes.

All included studies that scored high in the analysis domain were at high risk of bias. The risk of bias was unclear in 7 studies (22, 26, 27, 30, 33–35), and the remaining 12 studies (23–25, 28, 29, 31, 32, 36–40) were rated as high risk. At the time of the predictive model development study, the model was convincing when the events per-variable (EPV) ≥ 20, but two studies (22, 26) were unable to calculate the EPV, and one study (24) did not meet the requirement for the number of events in the study when the EPV was < 20 at the time of model development. Wang et al. (38) used a sample size of < 100 cases for the external validation of the prediction model. For continuous variables, the PROBAST evaluation entries advise against converting continuous variables to multicategorical variables, which can result in loss of information and reduced predictive accuracy of the model (43, 44). Seven studies (24, 27, 29, 30, 33, 34, 39) converted continuous predictors to categorical data without a reasonable explanation, 10 studies (22, 23, 25, 28, 31, 32, 35, 37, 38, 40) maintained the continuity of the predictors, and two studies (26, 36) did not report on the treatment of continuous variables. In terms of missing data, only 2 studies (31, 34) reported the direct exclusion of missing data, which could lead to selection bias and potentially negatively affect model performance during external validation (45), while the remaining studies did not report the treatment of missing data. One study (24) included indicators that were statistically significant at *P* < 0.05 on univariate analysis in a multifactorial Logistic regression analysis along with indicators that were not statistically significant at *P* > 0.05 for history of diabetes, concomitant with other catheterization-related complications, which the study did not justify, which may have led to an overfitting situation in the model that resulting in a model that performs well on the training set but has reduced generalization ability. One study (27) screened predictors only by univariate analysis during model development. Seven studies (26, 28, 32, 35–37, 39) did not consider model overfitting, underfitting, and optimality of model performance. None of the included studies provided information on the data complexity.

**Figure 3:**
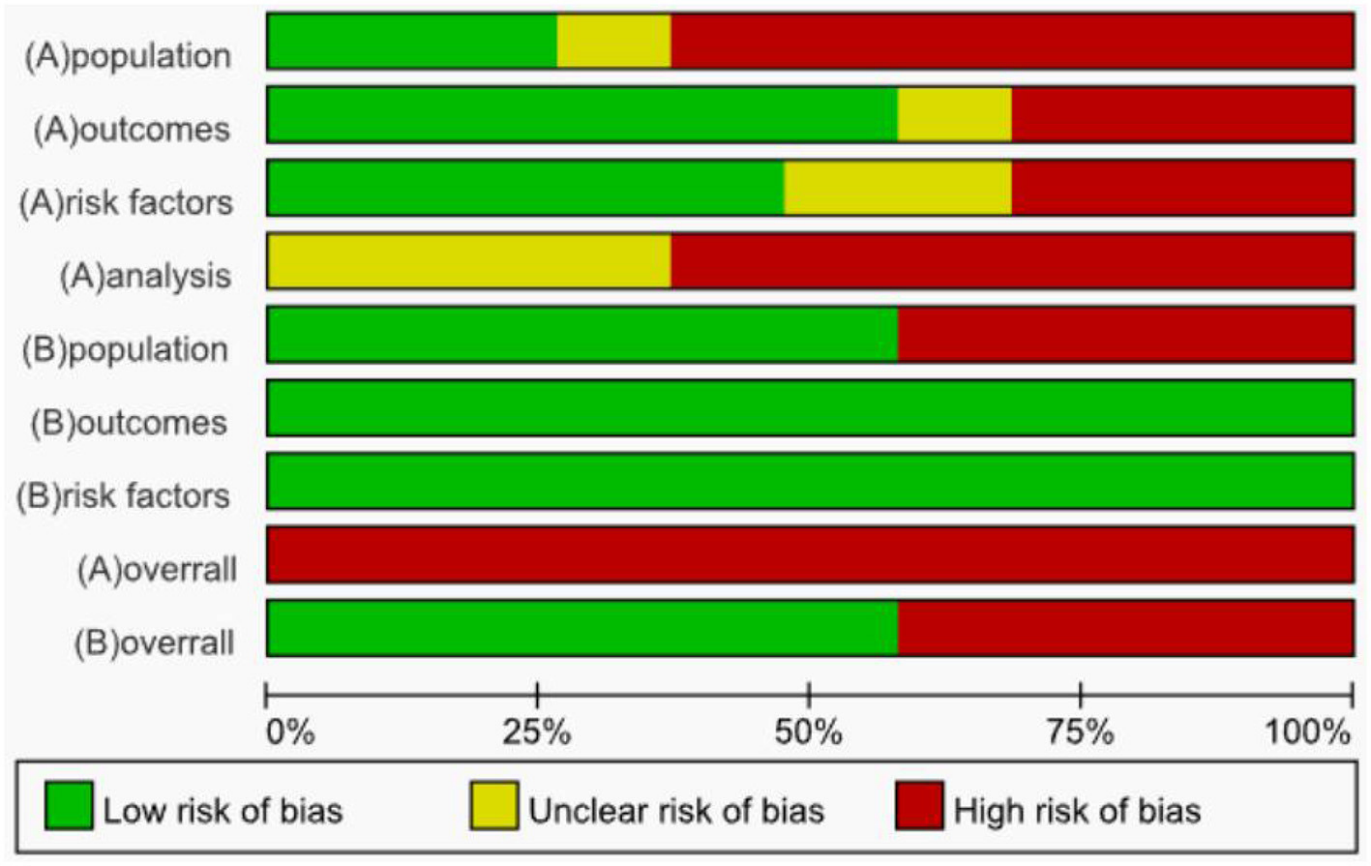
Summary results on risk of bias and applicability assessment (PROBAST).

In terms of applicability risk assessment, 11 studies (23, 25–33, 37) had good applicability in their overall assessment and 8 studies (22, 24, 34–36, 38–40) showed low applicability in the neighborhood of the study population. Six of the studies (24, 34–36, 38, 40) did not report the age of the included study population; 1 study (22) included study population aged between 7 and 86 years; and 1 study (39) included only patients with study population aged ≥65 years. All included studies showed good applicability in both the predictor and outcome neighborhoods. Detailed information on the risk of bias and applicability assessment is provided in Table 3 and Figure 3.

**Table 3:** Risk of bias and clinical applicability of included studies.

**Study ID**	**ROB**				**Applicability**			**Overall**	
	**Participants**	**Predictors**	**Outcome**	**Analysis**	**Participants**	**Predictors**	**Outcome**	**ROB**	**Applicability**
Wang, He, Chu, Chen, and Wang (22)	–	–	–	?	–	+	+	–	–
Dong, Zhang, Zhu, Sun, Xi, Li, and Gu (23)	–	?	+	–	+	+	+	–	+
Gao, Zhao, Yang, Sun, Hua, Wu, Wang, Gu, Zhou, and Bi (24)	+	+	+	–	–	+	+	–	–
Huang, Chen, Deng, Shi, and Shang (25)	–	?	+	–	+	+	+	–	+
Sun, Song, Zhang, and Song (26)	+	–	+	?	+	+	+	–	+
Zhang, Xie, Zhou, and Hao (27)	–	+	–	?	+	+	+	–	+
Zhou, Wang, Lu, Zhou, Liu, Dong, and Li (28)	–	+	+	–	+	+	+	–	+
Chen, Zhang, Li, Shi, and Gon (29)	–	–	–	–	+	+	+	–	+
Yang, Hua, Wu, Bi, Wu, Wang, Gao, Liang, and Wu (30)	+	–	?	?	+	+	+	–	+
Lin, Zhu, YihanZhang, Du, and Zhang (31)	–	+	+	–	+	+	+	–	+
Fu, Cai, Zeng, He, Bao, Lin, Lin, Hu, Lin, Huang, Zheng, Chen, Zhou, Lin, and Fu (32)	+	+	+	–	+	+	+	–	+
Peng, Wei, Li, Yuan, and Lin (33)	–	+	?	?	+	+	+	–	+
Liu, Xie, Sun, Wang, Yuan, Liu, Huang, Wang, Mo, Yi, Guan, Li, Wang, Li, Ma, and Zeng (34)	+	–	+	?	–	+	+	–	–
Song, Lu, Chen, Bao, Li, Li, Peng, Liu, Chen, Li, and Zhang (35)	?	–	–	?	–	+	+	–	–
Liu, Zhang, Xie, Wang, Xiang, Yue, Feng, Yang, Li, Luo, and Yu (36)	?	+	–	–	–	+	+	–	–
Hu, Wu, and Zhao (37)	–	?	–	–	+	+	+	–	+
Wang, He, Chu, and Xu (38)	–	+	+	–	–	+	+	–	–
Du, and Din (39)	–	?	+	–	–	+	+	–	–
Perek, Khatib, Izhaki, Khalaila, Brenner, and Horowitz (40)	–	+	+	–	–	+	+	–	–

PROBAST, Prediction model risk-of-bias assessment tool; ROB, risk of bias. + indicates low ROB/low concern regarding applicability; – indicates high ROB/high concern regarding applicability; and ? indicated unclear ROB/unclear concerns regarding applicability.

### 3.6 Meta-analysis of validation models included in the review

**Figure 4:**
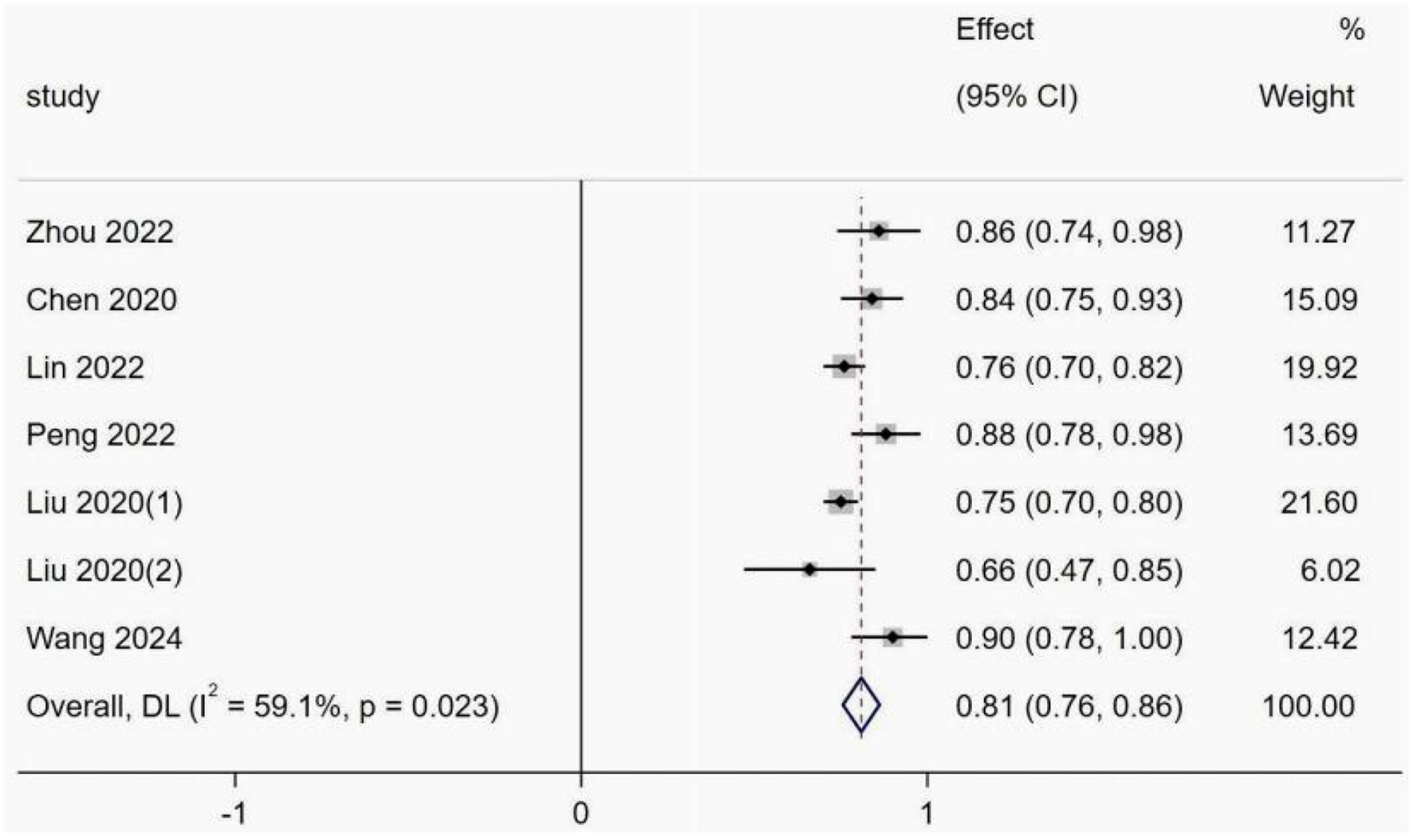
Forest plot of the random effects meta-analysis of pooled AUC estimates for six validation models.

This was due to the underreporting of model development details in the included studies and the fact that some models did not report validation set AUC values and ranges. Thus, only six studies met the inclusion criteria, of which one study (31) was time and geographically validated. Five studies (22, 28, 33, 35, 40) constructed predictive models of CRT risk in cancer patients based on machine learning methods all based on the same sample, and there are methodological differences between traditional and machine learning models, so that if models with too much difference are included, such as models containing both logistic regression and machine learning, the heterogeneity between the results increases, making it difficult to interpret the combined results, and may even lead to misleading conclusions. Therefore, only models constructed by logistic regression were included. Model discrimination was considered in all six included studies, with an AUC range of 0.470–1.000. The combined AUC value was 0.81 (95% confidence interval: 0.76–0.86) using a random effects model (Figure 4). The *I*^2^ value was 59.1% (*P* < 0.001), indicating a high degree of heterogeneity among the studies. However, subgroup analyses were not performed because the individual study types were different.

## 4 Discussion

CRT is one of the most common and risky complications in patients undergoing cancer catheterization (46). CRT is classified into four categories: deep vein thrombosis (DVT), thrombosed superficial phlebitis, asymptomatic thrombosis, and thrombotic catheter malfunction (3), with the majority of patients presenting with asymptomatic occult thrombosis (47). Risk assessment helps healthcare professionals identify the potential risk of CRT in cancer patients, stratify the risk of central venous CRT in cancer patients to target high-risk patients, optimize therapeutic decision-making, improve patients' and their caregivers' knowledge of risk management, facilitate communication, and ultimately improve patients' adherence to risk management behaviors. For early identification and intervention to reduce adverse outcomes, it is necessary to identify appropriate CRT risk prediction models that can be easily selected and applied by caregivers. This systematic review identified and critically evaluated 29 CRT prediction models reported in 19 studies, with a very small number of studies reporting poor predictive performance of the models. Most of the remaining models showed moderate to good predictive performance in internal or external validation. The model AUC values ranged from 0.470 to 1.00 in all included studies. However, based on the PROBAST inventory, all studies were considered to be at a high risk of bias, limiting the practical application of these models; the applicability of 11 of these studies is of high concern. Six validated models were included for Meta-analysis, and the combined AUC value was 0.81 (95% confidence interval: 0.76–0.86). However, there is a high degree of heterogeneity among models, which may be related to biological differences in cancer types or methodological differences. For example, significant differences in gene mutations, gene expression, epigenetic modifications, and metabolic characteristics of cancer cells in different regions, as well as differences in catheter types, can lead to high heterogeneity among models. In addition, variability in study design and analysis process can lead to higher heterogeneity among models. However, subgroup analysis was not performed in this study because of the small number of cancer types and catheter types included. Only risk prediction studies based on logistic regression models were included in this study for Meta-analysis, excluding machine learning models. This choice was mainly based on the widespread use of logistic regression models in clinical studies, the relative consistency of results reporting, and their good interpretability, properties that facilitate the merging of standardized Meta-analyses. However, this inclusion criterion may limit the applicability of our findings to more complex clinical scenarios. Logistic regression inherently assumes a linear relationship between predictor variables and outcomes (log odds), and complex interactions need to be predefined. In contrast, many complex clinical scenarios involve potentially non-linear associations and complex interaction effects between a large number of variables, and machine learning models are designed to be more adept at automatically capturing such complex patterns and better able to handle high-dimensional data. Future research should explore methodologies for integrating the predictive performance of machine learning models to more fully assess the value of risk prediction in driving the optimization of complex clinical protocols. In addition, during our model evaluation, we found that the TRIPOD statement (48) for transparent reporting of multivariate prediction models for individual prognosis or diagnosis was not well followed in several studies and that the lack of transparency creates a certain degree of potential risk of bias and uncertainty in the models. Eighteen of the included models were developed or validated based on Chinese patients, suggesting that prediction of the risk of central venous CRT development in patients with cancer is receiving increasing attention in China. Notably, more than 90% of the prediction models were published in the last 5 years, indicating that research in this area is not yet mature and there is a lack of accepted prediction models. Most of the studies included in our systematic review used cross-sectional and retrospective cohort study designs; however, prospective longitudinal cohort studies are preferred for the development or validation of predictive models (19, 20), and cross-sectional and retrospective studies tend to have some degree of bias toward predictors and outcome measures. Therefore, further updates or prospective longitudinal cohort studies with larger samples and more rigorous designs, multi-center external validation (temporal or spatial), and new predictive models with greater transparency of reporting are needed to support best clinical practice in the future.

All 19 studies included in the systematic evaluation were at high risk of bias, and differences in study design, statistical methods, and study quality may affect the AUC values of the studies to some extent. When the sample size is small, the modeling is not stable enough, and if the proportion of positive and negative samples in a study is severely imbalanced (e.g., rare disease testing), the AUC may be falsely high because the model tends to predict most categories. The AUC of prospective studies are usually more reliable than those of retrospective studies, but there may be selection bias. All six studies in this systematic evaluation were internally validated, and internally validated AUC may be overly optimistic; AUCs validated independently of external datasets are more convincing. Among the studies we included, 2 studies had direct deletion of missing data, and the remaining studies did not report the method of missing data handling; missing data deletion may lead to sample bias, resulting in a weakened ability of the model to test for the true presence of an effect; furthermore, AUC of the same type of study fluctuated depending on the time span (e.g., disease progression). In terms of statistical methods, improper data handling can have some impact on AUC; for example, most models (e.g., logistic regression, random forests) are unable to automatically recognize missing values and may treat them as exceptions (e.g., padded with zeros or extreme values), which may lead to incorrect feature segmentation and result in overfitting of the test set AUC. Directly removing missing values reduces the sample size, especially in small datasets, which decreases the stability of the model and leads to increased fluctuations in the AUC results. It is suggested that the use of multiple interpolation is considered the optimal solution regardless of the proportion of missing data (49). Continuous variables contain rich gradient information, which, if converted to categorical variables, may result in the model's inability to capture the non-linear or monotonic relationship between the continuous variable and the target variable, which may reduce the prediction accuracy. One study (50) reported that instead of categorizing continuous variables, it is better to keep them continuous. For example, we can use linear regression instead of two-sample *t*-tests. If there is a concern that linear regression does not provide a true representation of the relationship between the outcome and the predictor variables, some transformation (e.g., logarithmic transformation) can be explored. One study (24) included non-significant predictors, which may increase the risk of overfitting. Therefore, future studies should strictly follow the PROBAST criteria for model development, do a good job of handling missing data (multiple interpolation) to improve the quality of reporting, insist on a minimum EPV of 20 to enhance model robustness, and use multicenter and cross-regional external validation. From our systematic review of the included studies, it was clear that the predictors included in the models were relatively stable across studies. The predictors included factors related to the individual patient, the disease itself and its treatment, catheter placement, and laboratory markers. The top three retained variables were D-dimer, BMI, and Diabetes, and the predictors that appeared more frequently are informative for future research and nursing practice. The number of predictors for most of the models in the various studies ranged from 4 to 8, with one model containing only two predictors (30) reporting a C-index value of 0.71 (0.630–0.800), and the model was only internally validated with moderate predictive performance. The other model contained 10 predictors (24), which may be more complex and time-consuming to apply in clinical practice compared to other models with fewer predictors; the model was internally validated but reported a training set AUC value of only 0.794 (0.744–0.845). Although the other models had four to eight predictors, three studies (23, 35, 40) constructed models that were not validated, and four models (32, 34, 40) had an AUC value < 0.7 with low predictive performance. Therefore, in clinical work, medical staff should select prediction models according to the actual situation in clinical practice and recommend using a small number of predictors that are easy to measure and models with a high predictive performance. In the four included studies (23, 25, 37, 39), which were cross-sectional studies (23, 25, 37, 39), information related to whether the predictor assessment was blinded was not reported, resulting in a low risk of bias in the predictor domain. In addition, several studies converted continuous variables such as D-dimer level, BMI, and age to categorical data, but the cutoff point for categorization varied across studies and was not explained in the literature. Although the conversion of continuous variables to categorical data has some practical benefits, irrational categorization can bias predictive information to a certain extent, leading to lower predictive performance (49). Therefore, we suggest that the advantages and disadvantages should be carefully weighed when converting continuous variables into categorical variables. When continuous variables are converted to categorical variables, there may also be a loss of information due to improper selection of categorical boundaries. Therefore, we suggest that the pros and cons should be carefully weighed when converting continuous variables to categorical variables. Future studies can compare the effects of different treatments (e.g., continuous vs. categorical variables) on model results to assess robustness. The basis for variable treatment (e.g., whether the selection of critical thresholds was based on data distribution, clinical guidelines, or statistical optimization) should also be detailed in the paper.

We should do a better job of applying common predictors (e.g., D-dimer, BMI, diabetes) to the clinical setting. d-dimer is available through routine blood tests, and we can automate the integration of laboratory and clinical data, such as collaborating with laboratories to test for d-dimer to ensure standardization of tests (e.g., harmonization of assays) and automated access to results through the electronic health record (EHR). bMI and diabetes diagnosis need to be standardized. BMI and diabetes diagnosis need to be standardized, with BMI embedded in the EHR through automated calculator tools (generating BMI values in real time after entering a patient's height and weight) and mandatory measurements during the course of care (e.g., mandatory measurements on admission to the hospital). diabetes diagnosis can be confirmed based on a diagnostic code, reducing manual entry errors. However, there may be some challenges for rural areas, which may lack frequent testing equipment, making it more difficult to implement. In terms of model applicability, D-dimer-based models may be more suitable for cancers with a higher risk of thrombosis, such as lung cancer, but less sensitive for early-stage breast cancer, so the model should be selected according to the type of cancer in the clinical workup; also, D-dimer should be monitored dynamically: even if the risk is not initially assessed to be high, for patients with cancers with a high risk of thrombosis (especially when starting chemotherapy or new treatments), regular (e.g., monthly) Monitoring changes in D-dimer levels, which are consistently elevated or significantly elevated, is a strong predictor of thrombosis. In addition, standardized D-dimer testing in Chinese hospitals may not be applicable in resource-limited areas.

The studies included in this systematic review mainly used traditional logistic regression analyses to construct their models, and most of them achieved moderate to good performance. Five studies (26, 32, 36, 37, 39) developed models using ML methods, but three of these studies (26, 36, 37) incorporated the Seeley scale when constructing predictive models using ML methods. The Seeley Scale, originally developed by Professor Seeley at the Rush University Medical Center in the United States, is a risk assessment tool for predicting upper extremity venous thrombosis. The scoring system consisted of 5 items with corresponding values: 14 points for prolonged bed rest, 13 points for localized deep vein pressure, 10 points for smoking, 20 points for tubes inserted due to osteomyelitis, and 9 points for anticoagulation at home or during hospitalization. The total score was 66, with a score of ≥20 meaning that the patient was prone to upper limb venous thrombosis during hospitalization (42). If the scale was included in the construction of the model, it may have improved the model prediction performance to some extent and helped improve the accuracy of CRT prediction. However, when conducting clinical practice, it is necessary to assess and score the five entries within the Seeley scale, in addition to assessing the other predictors of the model, which makes the model contain too many predictors and may be too complex and time-consuming to use. Additionally, the presentation aspects of these models are unspecified, which may limit their use in clinical practice. Therefore, the selection of models should be based on clinical reality, and those that are easily applicable to the clinic should be considered. Two other studies (32, 40) selected two ML methods to construct models, two of which had low predictive performance, with AUC values < 0.7. In recent years, an increasing number of scholars have adopted ML methods to construct predictive models, which have the flexibility to capture complex associations in large unstructured data (especially healthcare data) as well as the complexity of modeling, compared with traditional regression methods. However, a systematic review showed that ML methods did not show a better performance advantage than logistic regression methods, implying that ML does not necessarily improve model performance (51). Nonetheless, there is still the potential for using ML methods to construct predictive models for large datasets.

The studies included in this systematic review focused on malignant neoplasms, breast cancer, lung cancer, hematological malignancies, and newly diagnosed acute myeloid leukemia. The performance of each predictive model may be poor when applied to other patients or settings (52). When applied to other patients or situations, CRT risk prediction models for cancer patients may vary across cancer types, catheter types, and medical settings. For example, CRT risk prediction models for lung cancer patients may lead to biased predictions when used to predict the risk of CRT in breast cancer patients because lung and breast cancers are two very different cancers in terms of pathologic type, growth mode, and metastatic pattern, and only 2 of the 19 studies we included (32, 33) developed models for predicting CRT risk in breast cancer patients, three studies (23–25) were models developed for predicting CRT risk in lung cancer patients, and most of the rest were CRT prediction models developed for mixed cancer populations. Second, in our systematic review, 14 studies (23–28, 30, 32, 33, 35–39) were PICC catheters, 4 studies (22, 31, 34, 40) were CVC catheters, and 1 study was (29) an IVAP catheter. Differences in central venous catheter types (CVC, PICC, and IVAP) in cancer patients may make the PICC thrombosis prediction model inapplicable to the prediction of CVC thrombosis, and PICC may be more likely to cause thrombosis than CVC due to the different weighting of factors such as catheter puncture time, tip design, and location of the catheter puncture, whereas the IVAP may be at lower risk due to complete implantation. In addition, standardized testing for D-dimer, for example, may not be feasible in resource-limited areas because of differences in healthcare settings. Therefore, we suggest that when selecting a model, the cancer type, central venous catheter type, and medical patient resource feasibility should be considered for selection. For rare cancer types, a central venous CRT risk prediction model for mixed cancer patients could be selected and validated to test the predictive performance of the model if necessary. Therefore, it is necessary to perform an internal or external validation of a model before applying it in clinical practice. Unfortunately, the models constructed by the studies included in this systematic review seldom carried out external validation, and the models constructed by three studies were not validated. The model developed by Liu et al. (34) in China was the only one that carried out validation at different times and locations, and most of the rest of the models were only internally validated, and the models that were validated in the original dataset may have optimistic predictive performance, but their generalization ability is not certain, and external generalizability is yet to be tested. In addition, in our systematic review, we found that researchers were keen to develop new central venous CRT prediction models for cancer patients, and few of them carried out validation and optimization of existing models. Therefore, future studies may prefer to carry out more external validation of existing models in new datasets and at different times and locations, optimize the models, improve their generalization ability, and assess the effectiveness and feasibility of the models in clinical practice, with the aim of providing reliable support for clinical decision-making.

When a clinical predictive model is deemed suitable for clinical practice, the form in which it is presented is also an important factor. Models are often presented in a format that is useful to the medical staff for clinical applications, including line graphs, scoring systems, web applications, and mobile apps (53). Among the included studies, the nomogram was chosen most often, followed by the scoring system. Although the format of model presentation facilitates the clinical development of applications, each presentation format has its advantages and disadvantages; therefore, the decision on model presentation should be based on the specific clinical context and the patient (54). In addition, thrombus evolution is a dynamic process, and a single follow-up visit is equivalent to a static investigation of the thrombus, which is insufficient to obtain dynamic information. Most of the studies included in this review were retrospective and cross-sectional investigative studies, and the central venous CRT reported for cancer patients were collected at a single follow-up visit, usually at the time of thrombosis, catheter removal, patient discharge, or a predetermined time point, and in some cases, asymptomatic thrombosis, and it is possible that the timing of the CRT may also have had an impact on the AUC values to some extent. Therefore, future prospective longitudinal studies should be conducted to predict the occurrence of CRT in stages according to time period (e.g., 1 week, 2 weeks, 1 month, 3 months, and 6 months after tube placement). Alternatively, a hybrid growth model (GMM) can be used to outline the trajectory of CRT, identify heterogeneity, and construct a dynamic column chart, which will help clinical practitioners screen high-risk patients, facilitate the implementation of risk stratification and effective thromboprophylaxis measures, and greatly facilitate the rational allocation of healthcare resources.

Although most of the studies included in our systematic review had more than moderate predictive performance, each model contained a different number of predictors, and individual models contained more complex predictor measures, such as some laboratory test indicators, which not only increase the expenditure of individual patients' healthcare costs, but also increase the burden of healthcare coverage in low- and middle-income countries. Therefore, low- and middle-income countries can choose models that are relatively good and have fewer predictors that are easy to obtain, and we recommend the model developed by Sun et al. (26), which has better predictive performance and predictors that are easy to obtain. High-income countries may choose to predict the model developed by Wang et al. (38), which has good predictive performance, and some predictors need to be examined in the laboratory to get the results, which is relatively more complicated and also increases the medical expenses, but as a high-income country may be easy to accept.

Currently, there is another issue that we should think about, most of the studies included in this systematic review did not mention whether thromboprophylaxis was taken for cancer patients, if thromboprophylaxis has been taken for patients before risk prediction, then using a specific risk prediction model to predict whether a patient develops CRT or not may lead to a decrease in the accuracy of the prediction model. Additionally, prophylaxis is often applied in a targeted manner to clinically judged high-risk patients, resulting in a reduction in outcome events for high-risk patients in the model development data set as a result of receiving prophylaxis, whereas low-risk patients may not have received the intervention. If this confounding factor is not adjusted for, models may incorrectly consider “receipt of prophylaxis” itself as a marker of low risk. If the rate or type of prophylaxis use in the validation cohort is different from that in the development cohort, the predicted probability of the model may not match the actual observed rate of thrombosis. When the prevalence of prophylaxis is low at the time of model development and widespread at the time of validation, the model will overestimate the risk. In addition, when thromboprophylaxis is used as an effective prophylactic measure and thromboprophylaxis is not performed when the patient is at high risk for CRT, it would be unethical to take thromboprophylaxis which would inevitably reduce the number of catheter-related thrombotic events in the study, and may even interfere with the risk factor-CRT correlation. Therefore, to address these types of issues, they need to be incorporated into the study design in the future.

## 5 Limitations

This study has several limitations. In terms of quality assessment of the included studies, the majority of the 19 included studies had a high risk of bias, which reduces the credibility of the systematic review to some extent, implying that there is an urgent need for the development of high-quality models for predicting CRT, and that the construction of the models needs to follow strict methodological guidelines. Second, only six internally and externally validated models were included in our meta-analysis due to the transparency of reporting and methodological differences of the included studies, which may have led to the inability to further discuss the heterogeneity among the studies as well as the low efficacy of study publication bias tests. However, these issues did not affect the assessment of the models. Future studies need to adopt more rigorous methods and more transparent reporting.

In terms of model performance and actual performance, 18 of the 19 studies were conducted in mainland China, which may somewhat limit the generalizability of the findings to Western populations due to differences in study populations, clinical protocols, genetics, and thromboprophylaxis strategies, and appropriate model adaptations may be required if these models are carried out for applications in other different regions. Future studies could analyze the potential adaptation or calibration of the models to specific populations, and could also develop risk prediction models for central venous catheter-associated thrombosis in patients with different cancers, which would be important for future research. Second, this review included four CRT risk prediction models constructed based on machine learning method, but because the risk of bias assessment tool for ML models has not yet been published, this review used the PROBAST standard for uniform risk of bias assessment, which may be biased in the quality of assessment. Our study excludes models with fewer than two predictors, which may leave out simple but practical models in resource-limited settings. Finally, this review only included published studies in English and Chinese, and did not include gray studies in other languages or unpublished, which may have some bias.

## 6 Conclusion

Nineteen studies with 29 models were included in this systematic evaluation to synthesize the quality and performance of the 29 CRT prediction models. The combined AUC of the 6 validated models was 0.81 (95% CI: 0.76–0.86), which was discriminating. There is a growing body of research on CRT prediction models for cancer patients to support medical decisions and strategies. Our review, despite reporting that several CRT prediction models performed moderately to well in internal datasets, their current clinical utility is limited due to high risk of bias, lack of external validation, and methodological inconsistencies. Future work should focus on updating models, performing external validation in different populations, and ensuring strict adherence to TRIPOD and PROBAST guidelines before implementation. Additionally, constructed models should be considered for long-term implementation and dissemination to maximize clinical utility and cost-effectiveness for patients.

## Data Availability

The original contributions presented in the study are included in the article/Supplementary material, further inquiries can be directed to the corresponding author.
