# Supplementary material for: Predictive model for CRT risk in cancer patients with central venous access devices: a systematic review and meta-analysis
Source: Front Med (Lausanne). 2025 Jun 27;12:1580920. doi: 10.3389/fmed.2025.1580920 (PMC12245904; doi:10.3389/fmed.2025.1580920)
Supplement: Supplementary file 1 [file Table_1.docx]

**Supplementary material 1. Search strategies and results for each database from inception to May 22, 2024**

| **Database** | **Search strategies** | **Results** |
| --- | --- | --- |
| PubMed | #1 "Neoplasms"[MeSH Terms] | 3971370 |
|  | #2 "Neoplasms"[MeSH Terms] OR "neoplasm*"[Title/Abstract] OR "neoplasia*"[Title/Abstract] OR "tumor*"[Title/Abstract] OR "cancer*"[Title/Abstract] OR "malignanc*"[Title/Abstract] OR "tumour*"[Title/Abstract] OR "neoplastic"[Title/Abstract] | 5177485 |
|  | #3 "Vascular Access Devices"[MeSH Terms] OR "Central Venous Catheters"[MeSH Terms] OR "catheterization，peripheral"[MeSH Terms] OR "Vascular Access Device*"[Title/Abstract] OR "Port Catheter*"[Title/Abstract] OR "Venous Reservoir*"[Title/Abstract] OR "Vascular Catheter*"[Title/Abstract] OR "Arterial Line*"[Title/Abstract] OR "Central Catheter*"[Title/Abstract] OR "central line"[Title/Abstract] OR "central vein catheter"[Title/Abstract] OR "central venous access catheter"[Title/Abstract] OR "central venous catheter*"[Title/Abstract] OR "central venous line"[Title/Abstract] OR "CVP"[Title/Abstract] OR "PICC"[Title/Abstract] | 33918 |
|  | #4 "Thrombosis"[MeSH Terms] OR "venous thrombosis"[MeSH Terms] OR "upper extremity deep vein thrombosis"[MeSH Terms] OR "venous thromboembolism"[MeSH Terms] OR "Thrombus"[Title/Abstract] OR "Blood Clot*"[Title/Abstract] OR "Thromboses"[Title/Abstract] OR "Venous Thrombos*"[Title/Abstract] OR "Deep Vein Thrombos*"[Title/Abstract] OR "catheter-related thrombosis"[Title/Abstract] OR "thrombotic"[Title/Abstract] OR "phlebothrombosis"[Title/Abstract] OR "venothrombo*"[Title/Abstract] OR "deep vein thrombophlebitis"[Title/Abstract] OR "DVT"[Title/Abstract] | 256103 |
|  | #5 "Nomograms"[MeSH Terms] OR "nomogram*"[Title/Abstract] OR "predict*"[Title/Abstract] OR "risk prediction"[Title/Abstract] OR "prediction model"[Title/Abstract] OR "risk score"[Title/Abstract] OR "assess"[Title/Abstract] OR "estimate"[Title/Abstract] | 3715555 |
|  | #6 #2 AND #3 AND #4 AND #5 | 222 |
| Embase | #1 'neoplasm'/exp OR 'tumor*':ti,ab OR 'tumour*':ti,ab OR 'neoplasia*':ti,ab OR 'neoplasm*':ti,ab OR 'neoplastic':ti,ab OR 'cancer*':ti,ab OR 'malignanc*':ti,ab | 7326566 |
|  | #2 'vascular access device'/exp OR 'central venous catheter'/exp OR 'central line':ti,ab OR 'central vein catheter':ti,ab OR 'central venous access catheter':ti,ab OR 'central venous line':ti,ab OR 'cvp':ti,ab OR 'picc':ti,ab OR 'vascular access device*':ti,ab OR 'port catheter*':ti,ab OR 'venous reservoir*':ti,ab OR 'vascular catheter*':ti,ab OR 'arterial line*':ti,ab OR 'central catheter*':ti,ab OR 'central venous catheter*':ti,ab OR 'catheterization，peripheral':ti,ab | 68696 |
|  | #3 'thrombosis'/exp OR 'vein thrombosis'/exp OR 'deep vein thrombosis'/exp OR 'venous thromboembolism'/exp OR 'upper extremity deep vein thrombosis'/exp OR 'thrombus':ti,ab OR 'Blood Clot*':ti,ab OR 'Thromboses':ti,ab OR 'Venous Thrombos*':ti,ab OR 'Deep Vein Thrombos*':ti,ab OR 'catheter-related thrombosis':ti,ab OR 'thrombotic':ti,ab OR 'phlebothrombosis':ti,ab OR 'venothrombo*':ti,ab OR 'deep vein thrombophlebitis':ti,ab OR 'DVT':ti,ab | 645399 |
|  | #4 'nomogram*':ti,ab OR 'predict*':ti,ab OR 'risk prediction':ti,ab OR 'prediction model':ti,ab OR 'risk score':ti,ab OR 'assess':ti,ab OR 'estimate':ti,ab | 5075825 |
|  | #5 #1 AND #2 AND #3 AND #4 | 703 |
| WOS | #1 TS=("Neoplasms" OR "neoplasm*" OR "neoplasia*" OR "tumor*" OR "cancer*" OR "malignanc*" OR "tumour*" OR "neoplastic") | 4640984 |
|  | #2 TS=("Vascular Access Devices" OR "Central Venous Catheters" OR "catheterization，peripheral" OR "Vascular Access Device*" OR "Port Catheter*" OR "Venous Reservoir*" OR "Vascular Catheter*" OR "Arterial Line*" OR "Central Catheter*" OR "central line" OR "central vein catheter" OR "central venous access catheter" OR "central venous catheter*" OR "central venous line" OR"CVP" OR "PICC") | 30733 |
|  | #3 TS=("Thrombosis" OR "venous thrombosis" OR "upper extremity deep vein thrombosis" OR "venous thromboembolism" OR "Thrombus" OR "Blood Clot*" OR "Thromboses" OR "Venous Thrombos*" OR "Deep Vein Thrombos*" OR "catheter-related thrombosis" OR "thrombotic" OR "phlebothrombosis" OR "venothrombo*" OR "deep vein thrombophlebitis" OR "DVT") | 284344 |
|  | #4 TS=("Nomograms" OR "nomogram*" OR "predict*" OR "risk prediction" OR "prediction model" OR "risk score" OR "assess" OR "estimate") | 7181633 |
|  | #5 #1 AND #2 AND #3AND #4 | 279 |
| The Cochrane Library | #1 MeSH descriptor: [Neoplasms] explode all trees | 125075 |
|  | #2 (neoplasm*):ti,ab,kw OR (neoplasia*):ti,ab,kw OR (tumor*):ti,ab,kw OR (cancer*):ti,ab,kw OR (malignanc*):ti,ab,kw OR (tumour*):ti,ab,kw OR (neoplastic):ti,ab,kw | 267335 |
|  | #3 #1 OR #2 | 280563 |
|  | #4 MeSH descriptor: [Vascular Access Devices] explode all trees OR MeSH descriptor: [Central Venous Catheters] explode all trees OR [Catheterization，peripheral] explode all trees OR (Vascular Access Device*):ti,ab,kw OR (Port Catheter*):ti,ab,kw OR (Venous Reservoir*):ti,ab,kw OR (Vascular Catheter*):ti,ab,kw OR (Arterial Line*):ti,ab,kw OR (Central Catheter*):ti,ab,kw OR (central line):ti,ab,kw OR (central vein catheter):ti,ab,kw OR (central venous access catheter):ti,ab,kw OR (central venous line):ti,ab,kw OR (CVP):ti,ab,kw OR (PICC):ti,ab,kw | 18390 |
|  | #5 MeSH descriptor: [Thrombosis] explode all trees OR MeSH descriptor: [venous thrombosis] explode all trees OR MeSH descriptor: [upper extremity deep vein thrombosis] explode all trees OR MeSH descriptor: [venous thromboembolism] explode all trees OR (Thrombus):ti,ab,kw OR (Blood Clot*):ti,ab,kw OR (Thromboses):ti,ab,kw OR (Venous Thrombos*):ti,ab,kw OR (Deep Vein Thrombos*):ti,ab,kw OR (catheter-related thrombosis):ti,ab,kw OR (thrombotic):ti,ab,kw OR (phlebothrombosis):ti,ab,kw OR (venothrombo*):ti,ab,kw OR (deep vein thrombophlebitis):ti,ab,kw OR (DVT):ti,ab,kw | 28385 |
|  | #6 MeSH descriptor: [nomograms] explode all trees OR(nomogram*):ti,ab,kw OR (predict*):ti,ab,kw OR (risk prediction):ti,ab,kw OR (prediction model):ti,ab,kw OR (risk score):ti,ab,kw OR (assess):ti,ab,kw OR (estimate):ti,ab,kw | 369816 |
|  | #7 #3 AND #4 AND #5 AND #6 | 101 |
| SinoMed | #1 "肿瘤"[不加权:扩展] OR "肿瘤"[常用字段:智能] OR "癌症"[常用字段:智能] | 7276526 |
|  | #2 "中心静脉导管"[不加权:扩展] OR "中心静脉导管"[常用字段:智能] OR "输液港"[常用字段:智能] OR "CVC"[常用字段:智能] OR "PICC"[常用字段:智能] OR "PORT"[常用字段:智能] | 69124 |
|  | #3 "血栓形成"[不加权:扩展] OR "血栓形成"[常用字段:智能] OR "血栓"[常用字段:智能] OR "导管相关性血栓"[常用字段:智能] OR "CRT"[常用字段:智能] OR "静脉血栓形成"[不加权:扩展] OR "静脉血栓形成"[常用字段:智能] OR "静脉血栓"[常用字段:智能] OR "静脉栓塞"[常用字段:智能] OR "上肢深静脉血栓形成"[常用字段:智能] | 542208 |
|  | #4 "列线图"[不加权:扩展] OR "风险预测"[常用字段:智能] OR "预测模型"[常用字段:智能] OR "风险评分"[常用字段:智能] OR "风险分层"[常用字段:智能] OR "列线图"[常用字段:智能] | 45343 |
|  | #5 #1 AND #2 AND #3 AND #4 | 35 |
| Wanfang Date | #1主题:("肿瘤" OR "癌症") | 2914769 |
|  | #2 主题:("中心静脉导管" OR "输液港" OR "CVC" OR "PICC" OR "PORT") | 276098 |
|  | #3 主题:("血栓形成" OR "血栓" OR "静脉血栓" OR "静脉栓塞" OR "上肢深静脉血栓形成" OR "导管相关性血栓" OR "CRT") | 396218 |
|  | #4 主题:("风险预测" OR "预测模型" OR "风险评分" OR "风险分层" OR "列线图") | 828832 |
|  | #5 #1 AND #2 AND #3 AND #4 | 43 |
| CNKI | #1 TKA=(肿瘤 + 癌症) | 1052811 |
|  | #2 TKA=(中心静脉导管 + 输液港 + CVC + PICC + PORT) | 179256 |
|  | #3 TKA=(血栓形成 + 血栓 +静脉血栓 + 静脉栓塞 + 导管相关性血栓 + 上肢深静脉血栓形成 + CRT) | 163191 |
|  | #4 TKA=(风险预测 + 预测模型 + 风险评分+ 风险分层 + 列线图) | 164693 |
|  | #5 #1 AND #2 AND #3 AND #4 | 29 |
| VIP | #1M=(肿瘤 OR 癌症 ) | 721815 |
|  | #2M=(中心静脉导管 OR 输液港 OR CVC OR PICC OR PORT) | 63387 |
|  | #3M=(血栓形成 OR 血栓 OR 静脉血栓 OR 静脉栓塞 OR 上肢深静脉血栓形成 OR 导管相关性血栓 OR CRT) | 104322 |
|  | #4M=(风险预测 OR 预测模型 OR 风险评分 OR 风险分层 OR 列线图) | 78039 |
|  | #5 #1 AND #2 AND #3 AND #4 | 16 |
